# Sequential chemotherapy and intensity-modulated radiation therapy in the management of locoregionally advanced nasopharyngeal carcinoma: Experience of 370 consecutive cases

**DOI:** 10.1186/1471-2407-10-39

**Published:** 2010-02-10

**Authors:** Shaojun Lin, Jiade Jay Lu, Lu Han, Qisong Chen, Jianji Pan

**Affiliations:** 1 Department of Radiation Oncology, Cancer Hospital of Fujian Medical University, Fuzhou, Fujian, PR China; 2 Department of Radiation Oncology, National University Cancer Institute, National University Health System, National University of Singapore, 5 Lower Kent Ridge Road, Singapore 119074, Singapore

## Abstract

**Introduction:**

To investigate the outcome of locoregionally advanced nasopharyngeal carcinoma (NPC) treated with intensity-modulated radiation therapy (IMRT) after induction chemotherapy, with or without concomitant chemotherapy.

**Methods:**

Between August 2003 and March 2007, 370 patients with locoregionally advanced NPC were treated with IMRT. Presenting stages were stage IIB in 62, stage III in 197, and stage IVA/B in 111 patients. All patients except for 36 patients with cervical lymphadenopathy of 4 cm or less in diameter received 2 cycles of cisplatin-based neoadjuvant chemotherapy. Forty-eight patients received cisplatin-based concurrent chemotherapy as well.

**Results:**

With a median follow-up time of 31 months (range 5 to 61 months), the 3-year local control, regional control, metastasis-free survival (MFS), disease-free survival (DFS) and overall survival (OS) rates were 95%, 97%, 86%, 81% and 89%, respectively. Multivariate analyses revealed that both age (≤ 60 vs. >60) and N-classification are significant prognosticators for OS (P = 0.001, hazard ratio [HR] 2.395, 95% confidence interval [CI] 1.432-4.003; P = 0.012, hazard ratio [HR] 2.614, 95% confidence interval [CI] 1.235-5.533); And N-classification is the only significant predicative factor for MFS (P = 0.002, [HR] 1.99, 95% CI 1.279-3.098). T-classification and concurrent chemotherapy were not significant prognostic factors for local/regional control, MFS, DFS, or OS. Subgroup analysis revealed that concurrent chemotherapy provided no significant benefit to IMRT in locoregionally advanced NPC, but was responsible for higher rates of grade 3 or 4 acute toxicities (50% vs. 29.8%, P < 0.005). No grade 3 or 4 late toxicity including xerostomia was observed. However, two patients treated with IMRT and neoadjuvant but without concurrent and adjuvant chemotherapy died of treatment related complications.

**Conclusion:**

IMRT following neoadjuvant chemotherapy produced a superb outcome in terms of local control, regional control, MFS, DFS, and OS rates in patients with stage IIB to IVB NPC. Effective treatment strategy is urgently needed for distant control in patients diagnosed with locoregionally advanced NPC.

## Background

Nasopharyngeal cancer (NPC) is the most commonly diagnosed head and neck malignancy in Southeast Asia, and radiation therapy is its mainstay treatment modality. For locoregionally-advanced diseases, concurrent chemoradiation therapy is considered the standard of treatment. Chemotherapy delivered concurrently with conventional radiotherapy has been shown to improve local control, disease-free survival, as well as overall survival rates for NPC patients with T2B, T3 or T4 diseases, or with neck lymphadenopathy [1-5].

Intensity-modulated radiation therapy (IMRT) enables the delivery of higher radiation dose to the primary disease and neck metastases while sparing the organs/tissues at risk (OARs) thereby enhancing the therapeutic ratio. It has been accepted as a more advantageous treatment technique as compared to conventional radiation for NPC. The clinical advantages of IMRT in the treatment of NPC with respect to both disease control and adverse-effect profiles have been repeated demonstrated [6-10]. The local control and regional control were particularly encouraging after IMRT, exceeding 95% in some of the previous reports [7-10].

Although considered essential for patients with locoregionally advanced NPC, the therapeutic value of chemotherapy given concurrently with IMRT and the optimal strategy of combining utilization of chemotherapy and IMRT have not been sufficiently addressed. The rationale of concurrent chemotherapy with IMRT in the management of NPC was largely derived from experience with conventional radiotherapy. With local and regional control rates both approximating 95% after IMRT, it is reasonable to question whether chemotherapy offers equal therapeutic benefits to all subgroups of patients with locoregionally advanced NPC, i.e., patients with T2B, T3, T4, and/or limited N+ classification such as N1/2 disease. The aim of this report is to address the treatment outcome and to elucidate the efficacy of concurrent chemotherapy in NPC treated with IMRT by analyzing the outcome of a relatively large group of patients with locoregionally advanced NPC treated uniformly with IMRT following neoadjuvant chemotherapy, with or without concurrent chemotherapy.

## Methods

### Patients and pretreatment evaluation

Between August 2003 and March 2007, 382 histologically diagnosed non-metastatic nasopharyngeal carcinoma (NPC) patients were treated primarily with intensity-modulated radiation therapy (IMRT) according to an IRB approved institutional treatment protocol at Fujian Provincial Tumor Hospital. Pretreatment evaluation consisted of a complete history and physical examination, flexible fiberoptic endoscopic examination, complete blood counts, blood chemistries, urinalysis, chest X-ray, electrocardiogram, computed tomography (CT) scans of the nasopharynx and neck, bone emission computed tomography (ECT) scans, ultrasound of liver and abdominal lymph nodes, and dental evaluation. Magnetic resonance imaging (MRI) scans of the head and neck were performed instead of CT in all patients diagnosed after July 2005. Positron emission tomography (PET) scans, CT scans of the chest and abdomen were optional and were performed when clinically indicated.

Tumors were staged according to the AJCC (1997) cancer staging classification [11]. Patients who had evidence of distant metastasis were not eligible for this treatment protocol. Ten (10) patients who had early stage (i.e., T1N0M0 or T2N0M0) diseases, as well as 2 patients who did not complete the planned radiation using IMRT to definitive dose were excluded from this analysis. Characteristics of the remaining 370 patients with stage IIB to IVB NPC are listed in Table 1.

**Table 1:** Patient characteristics

	Number of patients		
	With concurrent chemotherapy	Without concurrent chemotherapy	*P *Value
**Age, year**			P = 0.1
≥ 60	44	263	
<60	4	59	
**Gender**			P = 0.02*
Male	43	238	
Female	5	84	
**Histology**			N/A
WHO Type I	0	2	
WHO Type II	2	0	
WHO Type III	46	319	
**Stage**			P = 0.02*
IIB	2	60	
III	36	161	
IVA/B	10	101	
**KPS**			P = 0.77
90	42	283	
80-90	6	36	
70-80	0	3	

* Significantly lower proportions of male and patients with stage IVA/B received concurrent chemotherapy as compared to female and patients with stage III disease.

### Radiation Therapy Techniques

The techniques of planning and delivery of IMRT were described previously [10]. Briefly, all patients were immobilized in the supine position with thermoplastic masks. CT scans with IV contrast using 3 mm slices from the head to the level of 2 cm below the sternoclavicular joints were performed for planning. CT scan only were used for target delineation prior to July 2005, and planning CT scans with MRI-CT fusion using co-registration software (Oncentra Materplan^® ^version 1.5) were performed after July 2005 for all patients.

**Figure 1:**
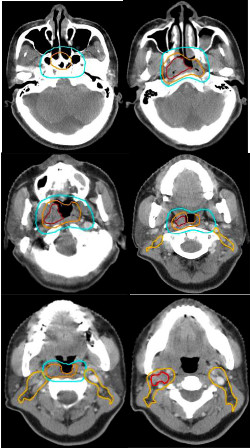
**Selected CT slides for demonstrating the delineation of target volumes**. (Red line: GTV-P and GTV-N; Orange line: CTV-1; Blue line: CTV-2; Yellow line: CTV-N).

The primary and nodal gross tumor volumes (GTV-P and GTV-N) included all gross diseases visualized on CT and/or MRI. The high-risk clinical tumor volume (CTV-1) included GTV plus 5-10 mm margin and encompassed the entire nasopharyngeal mucosa plus 5 mm submucosal volume. CTV-2 was designed for potentially involved regions included the nasopharyngeal cavity (limited only to the posterior part of nasal cavity), maxillary sinus (limited to 5-mm anterior to the posterior nasal aperture and maxillary mucosa), pterygopalatine fossa, posterior ethmoid sinus, parapharyngeal space, skull base, anterior third of clivus and cervical vertebra, inferior spheniod sinus and cavernous sinus, and included the retropharyngeal lymph nodal regions from the base of skull to cranial edge of the second cervical vertebra. The CTV of the neck nodal regions (CTV-N) included level II, III, IV, V, which were outlined according to the recommendation by the RTOG/EORTC CTV delineation protocol for head and neck malignancies (Figures 1) [12]. The planning target volume (PTV) was created based on each volume with an additional 3-mm margin, allowing for setup variability. Critical normal structures including the brainstem, spinal cord, parotid glands, optic nerves, chiasm, lens, eyeballs, temporal lobes, temporomandibular joints, mandible, hypophysis were contoured and set as OARs during optimization.

The primary tumor and upper neck above the bottom of hyoid bone (level II and upper level V) was treated with IMRT techniques using seven coplanar beams, separated at 51° apart. Inverse treatment planning using the Plato treatment planning software systems (RTS^® ^version 2.6.4) was used for all patients. A mono-isocentric technique was used for all these fields with the isocenter set at the center of GTV-P. The radiation dose prescribed in the protocol evolved: Before July 2006, a total dose of 66 Gy 30 fraction at 2.2 Gy/fraction to the GTV-P and GTV-N, 60 Gy at 2 Gy/fraction to the CTV-1, 54 Gy at 1.8 Gy/fraction to CTV-2 and CTV-N were prescribed; After July 2006, a total dose of 69.75 Gy in 31 fraction at 2.25 Gy/fraction to the GTV-P, 66.65 Gy at 2.15 Gy/fraction to the GTV-N, 60.45 Gy at 1.95 Gy/fraction to the CTV-1, 55.8 Gy at 1.8 Gy/fraction to CTV-2 and CTV-N were prescribed. This change of prescription was approved by our institution for the protocol. All patients were treated with one fraction daily, 5 days per week. The dose received by each OAR should less than its tolerance limit according to the RTOG 0225 protocol. The volume of each parotid received >26 Gy dose should no more than 50%. A conventional technique (AP field) was used for the lower neck (level III, level IV, lower level V). It was treated with 28 fractions of 1.8 Gy/fraction, to a total dose of 50.4 Gy with a depth of 3 cm from the anterior surface. The IMRT field was matched with the AP field with a split-beam technique, as described previously [13]. Treatment was delivered with a computer-controlled auto sequence multi-leaf collimator (MLC) on a linear accelerator (Eleckta Precise^®^) equipped with 40-leaf MLC.

Seventy-three (73) patients received boost treatment after the planned course of IMRT because of gross residual disease (i.e., less than 100% resolution of the primary disease) observed on follow-up CT or MRI or during nasopharyngoscopy. Among these patients, 20 were treated with brachytherapy boost with a dose of 6-12 Gy (2-3 Gy/fraction, 2 fracion/day), and external beam radiation were used in the remaining 53 patients to a total dose of 6-12 Gy at 2.2-3 Gy per daily fraction. Twenty-eight (28) patients received boost irradiation (4.5-10 Gy) to the cervical lymph nodes for residual adenopathy of more than 1 cm in diameter on follow-up CT or MRI.

### Chemotherapy

To prevent disease progression during treatment planning and waiting prior to the start of definitive treatment using IMRT, neoadjuvant chemotherapy was given to 308 patients with AJCC stage III-IVB diseases and 26 stage IIB patients with neck adenopathy of more than 4 cm in diameter. Thirty-six patients whose neck adenopathy measured 4 cm or less in diameter did not receive neoadjuvant chemotherapy. Neoadjuvant chemotherapy consisted of 2 cycles of cisplatin (80 mg/m^2 ^IV in days 1-3) plus 5-FU (800 mg/m^2 ^IV in d1-d5) or paclitaxel (135 mg/m^2 ^IV on the first day) spaced 2 weeks apart prior to the initiation of IMRT treatment, and IMRT started within 1 week after the second cycle of chemotherapy. Concurrent and adjuvant chemotherapy was not part of this standardized protocol for NPC treatment; nevertheless, concurrent cisplatin-based chemotherapy (cisplatin 80-100 mg/m^2 ^given over day 1-3 of each 21-day cycle) was given to 48 patients at the discretion of the attending radiation oncologists. In addition, adjuvant cisplatin-based chemotherapy was given to 71 patients at the discretion of the attending radiation oncologists.

### Follow-up

All patients were evaluated weekly during radiation therapy, and were required to be followed-up by their attending radiation oncologist after the completion of their treatment every 3 months in the first 2 years, every 6 months from year 2 through year 5, and annually thereafter. Each follow-up included a complete examination, basic serum chemistry, chest X-ray, and ultrasound of liver and abdomen. Flexible fiberoptic endoscopy was performed at every visit after treatment. MRI of the head and neck areas was performed every 6 months. Treatment induced toxicities were accessed and scored according to the RTOG radiation morbidity scoring criteria at each follow-up [14].

### Statistics

The actuarial local/regional control, metastatic-free survival, disease-free survival, and overall survival rates were calculated by the Kaplan-Meier method. The duration of time to locoregional failure and distant metastasis was measured from the date of the completion of radiation therapy (including boost irradiation) until documented treatment failure. The duration of overall survival was calculated from diagnosis until death or until death or until the date of the last follow-up visit for patients still alive.

Log-rank test was used to detect the significant difference in survivals between different prognostic groups. Multivariate analysis using the Cox proportional hazard model was performed for the aforementioned endpoints to define independent predictors among various potential prognostic factors.

## Results

### Treatment outcomes

The median follow-up time for the entire group was 31 months (range 5 to 61 months). At the time of their last follow-up, 15, 10, and 46 patients had developed local, regional, and distant metastasis, respectively, including 5 cases with both distant and local/regional recurrences. The 3-year estimated local control, regional control, metastasis-free survival (MFS), disease-free survival (DFS) and overall survival (OS) were 95%, 97%, 86%, 81% and 89%, respectively.

Thirty-eight (10.3%) patients had deceased at the time of this analysis: 22 patients died from distant metastasis, 7 died from progression of locoregional disease after recurrence. One patient died from late complication caused by radiation therapy (septicemia secondary to untreated infection occurred in the post-nasal space) at 12 months, and 1 patient died of bleeding from nasopharynx at 6 months after the completion of treatment. Both deaths were considered as treatment related. In addition, 3 patients died from co-morbidities unrelated with NPC, 1 died from traffic accident, and the causes of death of 3 additional cases were not reported.

Among all patients with local or regional failures, 1, 4, 8, and 8 had T1, T2, T3, or T4 diseases, and 0, 11, 10, 0 had N0, N1, N2, N3 diseases, respectively. Therefore, 50%, 3.3%, 5.8%, and 19.1% of patients with T1 to T4 diseases, and 0%, 8.1%, 5.3%, and 0% of patients with N0 to N3 diseases experienced local or regional recurrence, respectively. The regional control of patients with N0, N1, N2, and N3 diseases were 100%, 96.7%, 96.9%, and 100%, respectively (P = 0.62). No significant correlation can be found between T- and N-classifications with local and/or regional recurrences.

**Figure 2:**
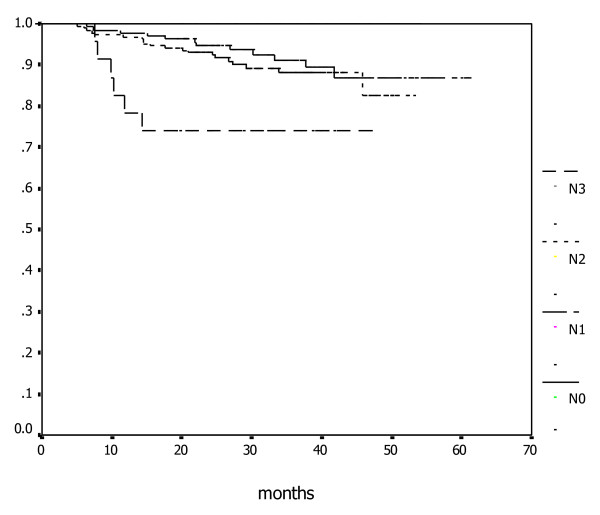
**Kaplan-Meier estimate of overall survival according to N-classifications**.

**Figure 3:**
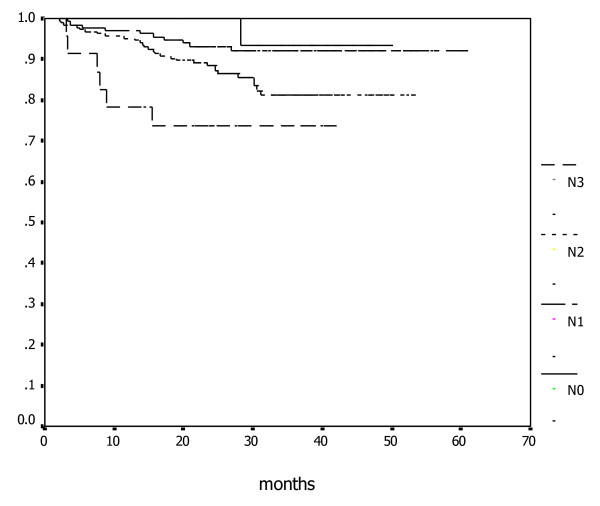
**Kaplan-Meier estimate of metastatic-free survival according to N-classifications**.

Among all patients with distant metastasis, 0, 12, 19, and 15 had T1, T2, T3, or T4 diseases, and 1, 10, 29, 6 had N0, N1, N2, N3 diseases, respectively. The associations between cervical nodal status and MFS, DFS, and OS rates were presented in Table 2. Figures 2 and Figure 3 illustrate the association between OS and MFS with neck node status (N-classification).

**Table 2:** Three-year overall survival rates according to patients' N-classification

	N0	N1	N2	N3	P Value
DFS	93.3	84.9	76.3	73.9	0.07
MFS	93.3	92.2	81.2	73.7	0.007
OS	100	91.2	88	73.9	0.006

Abbreviations: MFS: metastatic-free survival rate; DFS: disease-free survival rate; OS: overall survival rate

No significant association could be detected between the outcome including MFS, DFS, or OS and T-classification. No significant improvement in local and/or regional control rates as well as 3-year MFS, DFS or OS could be detected between patients received IMRT with or without concurrent chemotherapy (Tables 3 and 4). Analyses for MFS, DFS, or OS for patients with cervical lymphadenopathy or extensive lymph adenopathy also revealed no significant difference between patients treated using IMRT with or without concurrent chemotherapy (Tables 5 and 6).

**Table 3:** Local and/or regional control of disease treated with or without concurrent chemotherapy

	No concurrent chemotherapy	With concurrent chemotherapy	P Value
Local control	94.7%	97.8%	0.62
Regional control	97.4%	96.8%	0.96
Locoregional control	93%	94.6%	0.8

**Table 4:** Three-year survival rates in patients treated with or without concurrent chemotherapy

	No concurrent chemotherapy	With concurrent chemotherapy	P Value
MFS	85.8%	84.5%	0.65
DFS	80.3%	82.6%	0.48
OS	89.2%	89.1%	0.82

**Table 5:** Three-year survival in the 346 patients with N+ disease treated with vs. without concurrent chemotherapy

	No concurrent chemotherapy	With concurrent chemotherapy	P Value
MFS	82.5	83.7	0.50
DFS	79.3	81.7	0.66
OS	88.3	88.5	0.85

**Table 6:** Three-year survival in the 210 patients with N2-3 disease treated with or without concurrent chemotherapy

	No concurrent chemotherapy	With concurrent chemotherapy	P Value
DFS	74.6	81.7	0.24
MFS	79.3	84	0.22
OS	85.8	89.3	0.71

### Acute and late toxicity

All 370 patients tolerated the treatment well and completed the planned therapy. The most commonly observed severe (i.e., grade 3 or 4) acute toxicities included grade 3 mucositis, skin desquamation, and leucocytopenia, which occurred in 89(27.5%), 15(4.6%), and 19(5.9%) patients, respectively. One patient presented grade 4 leucocytopenia. The total incidence of grade 3 or 4 acute toxicities in patients received IMRT plus concurrent chemotherapy was 50%, significantly higher than those received IMRT alone (29.8%) (P < 0.005).

The most commonly observed late effect was xerostomia. The severity of xerostomia was evaluated at 3, 6, 12, and 24 months after radiotherapy. A total of 5.1% and 94.9% patients complained of grade 1 or 2 xerostomia, respectively, at 3 months. The severity of xerostomia diminished over time, and the detectable xerostomia at 24 months was 7.8%. No grade 3 or 4 xerostomia or other late-effects was detected. Two patients developed infection or bleeding followed by necrosis in the post-nasal space after treatment at 12 and 6 months respectively. Both patients received radiation therapy after neoadjuvant chemotherapy without concurrent and adjuvant chemotherapy. And both patients declined medical attention and deceased, and were considered as grade V toxicity.

### Prognostic factors

The value of various potential prognostic factors include age, gender, stage, use of boost and use of chemotherapy on predicting local control, MFS, DFS, and OS rates were evaluated in multivariate analysis. The number of local or regional failures was too small to allow meaningful analysis, and no prognostic factor was significant for local or regional control.

T-classification was not a significant prognostic factor for local/regional control, MFS, DFS, or OS. N-classification and age (≤ 60 vs. >60) were found to be the independent predictors for overall survival (P = 0.001, hazard ratio [HR] 2.395, 95% confidence interval [CI] 1.432-4.003; P = 0.012, hazard ratio [HR] 2.614, 95% confidence interval [CI] 1.235-5.533); furthermore, N-classification was a significant prognostic factor for metastasis-free survival (P = 0.002, [HR] 1.99, 95% CI 1.279-3.098).

Concurrent and adjuvant chemotherapy provided no significant additive effect on local/regional control, MFS, DFS, and OS rates in this group of patients with locoregionally advanced NPC, regardless of their T-classification or nodal status, definitively treated with IMRT in multivariate analysis (P > 0.05).

## Discussion

Concurrent chemoradiation therapy has been considered as the standard modality of locoregionally advanced NPC [15]. Although the efficacy of chemotherapy used in concomitant with conventional radiation therapy has been repeatedly proven [1-5], the additive value of concurrent chemotherapy on local/regional control and survival rates for locoregionally advanced NPC treated with IMRT is largely unknown. The results of the current study demonstrated that the local control rate of patients with T2b-T4 NPC was 95%, and the regional control of patients with cervical lymphadenopathy, regardless of its extent, was 97% at three-years after IMRT following cisplatin-based neoadjuvant chemotherapy. The metastasis-free survival (MFS), disease-free survival (DFS), and overall survival (OS) rates were 86%, 81%, and 89%, respectively, indicating that distant metastasis remained the major cause of treatment failure. Furthermore, multivariate analyses revealed that no significant prognostic factor including T-classification and concurrent chemotherapy were identified for local and/or regional control after neoadjuvant chemotherapy plus IMRT for this group of patients with locoregionally advanced NPC. Although patients' age was significant for predicting the overall survival rate, the N-classification was the only significant prognostic factor for both MFS and OS. Our data suggested that the use of concurrent chemotherapy added little value to local and distant disease control for patients with locoregionally advanced NPC treated with neoadjuvant chemotherapy and IMRT.

Despite of the proven efficacy of chemotherapy delivered concurrently with conventional radiation, the combined treatment strategy comes with substantial adverse-effects. In the pivotal Intergroup 0099 trial, grade 3 and 4 adverse effects in patients treated with concurrent chemoradiation therapy nearly doubled those received irradiation only. In addition, 37% patients discontinued concurrent chemoradiation therapy prematurely due to intolerance to combined treatment [1]. Similar results on treatment-induced complications have been documented in randomized and retrospective studies [2-4,16]. Obviously, treatment induced side effects could impede the utilization of concurrent chemoradiation, thus adversely affect the treatment outcome for patients with locoregionally advanced NPC. Relatively favorable outcome in terms of both local/regional control and overall survival were observed in the current study. These results indicated that concurrent chemotherapy that significantly increases the probability and severity of treatment-induced side effects might not be essential in the treatment of NPC if IMRT is used. We consider our results important as it suggests that omission of concurrent chemotherapy maybe possible, and effective disease control in the primary area and cervical nodes can be achieved with improvement in radiation technology and utilization of sequential chemotherapy.

Chemotherapy provides two purposes in the treatment of NPC: to improve local and regional control by improving the radio-sensitivity, and to further improve overall survival by controlling subclinical distant metastatic foci. In the aforementioned Intergroup study reported by Al-Sarraf, patients with stage III or IV NPC staged according to the 1992 AJCC staging system (including T2BN1M0 disease staged according to the revised 1997 system) were treated with either radiotherapy alone or concurrent chemoradiation therapy after randomization. The results of this pivotal study revealed that concurrent chemoradiation therapy significantly improve the local control, disease-free and overall survival rates [1]. These favorable results were subsequently confirmed by a number of randomized clinical trials completed in Asia and a meta-analysis [2-5]. However, for patients with stage IVB diseases, the rate of distant metastasis approached 40% despite aggressive chemotherapy [4]. Furthermore, previously reported results from randomized trials on neoadjuvant or adjuvant chemotherapy using similar chemotherapy regimen, dosage, and intensity failed to demonstrate any survival benefit from chemotherapy [5,17]. Thus, it is reasonable to speculate that improved local and/or regional control was the underlying mechanism of the improved survival for patients treated with concurrent chemoradiotherapy. The 3-year overall survival rate of 89% without concurrent chemotherapy in the current series further suggested that improved local and regional control rates could result in an improved overall survival.

With the increasing utilization of IMRT in the treatment of NPC, improved outcome in terms of local and regional controls had been repeatedly demonstrated. The three-year local/regional control and overall survival rates were reportedly in the range of 90%-95% and 80%-85%, respectively [7-10]. Improved local and regional controls were anticipated with improved dose coverage of gross and clinical tumor volumes. However, conventional technique was used in the aforementioned randomized trials on current chemoradiotherapy, and all patients with locoregionally advanced NPC reported in the IMRT trials received concurrent chemotherapy. Therefore, the effect of chemotherapy on locoregional control remained uncertain in NPC patients definitively treated with IMRT. Our results demonstrated that for patients with stage IIB to IVB diseases, neoadjuvant chemotherapy followed by IMRT produced a superb outcome, and no difference was detected when compared to those treated with concurrent chemoradiation. Our DFS and OS rates reported exceed those of patients treated with concurrent chemoradiation reported in randomized clinical studies including the Intergroup 0099 trial. Although direct comparison of results from different clinical trials is not feasible, substantial improvement from the superb local and regional control rates of >95% may require a paradigm shift in the current chemotherapy strategy.

As far as we know, this is the first study to address the outcome for locoregionally advanced NPC treated using IMRT with or without concurrent chemotherapy. Although no difference was shown between patients treated with or without concurrent chemotherapy, we consider our results far from conclusive. Firstly, among the 370 patients included in the current analysis, less than 50 received concurrent chemotherapy. The imbalance in these two subgroups of patients made the interpretation of our results difficult. However, since the 3-year local and regional control rates equaled 95% and 97%, respectively for patients treated without concurrent chemotherapy, detecting significant improvement with a magnitude of 1%-2% from such superb outcome is unlikely even with a substantial number of patients in both arms. In addition, a significant improvement in the 3-year overall survival from 90% is also unlikely with an improved balance between the two arms. Secondly, the median follow-up time of the current series was approximately 31 months. Although longer follow-up is desired to document the long-term outcome, as majority of local/regional recurrences occur in the first 24 months after the complication of radiation therapy [18-20], a median follow-up of 31 months in our series suggested that the true incidence of local and/or regional recurrence might approximate our findings.

Neoadjuvant chemotherapy was used in all patients with locoregionally advanced NPC in our study. The purpose of this strategy was to prevent disease progression during our long waiting period. While such a measure maybe necessary to prevent adverse outcome due to limited resource, the addition of chemotherapy prior to the start of IMRT may complicate interpretation of local and regional control outcome. Neoadjuvant chemotherapy is effective in reducing local recurrence without affecting overall survival. However, as all patients with stage III, stage IVA/B diseases, or with cervical adenopathy of >4 cm in diameter received chemotherapy prior to the start of IMRT, we consider neoadjuvant chemotherapy not a confounder of this analysis for the effect of concurrent chemotherapy. However, whether neoadjuvant chemotherapy could be omitted in the treatment of patients with locoregionally advanced NPC using IMRT awaits further investigations.

The retrospective nature of the study certainly served as an inherited and fundamental pitfall of the current study. In addition, it is important to note the significant imbalance between the patient groups treated with or without concurrent chemotherapy: higher proportion of female patients and higher proportion of patients with stage III NPC received concurrent chemotherapy, as compared to male and patients with stage IVA/B disease. Although such imbalances served a substantial shortcoming for a retrospective study, it is equally importantly to note that more patients with poorer prognostic factors (male gender and higher stage) were treated without concurrent chemotherapy, which is considered a less aggressive approach. Subgroup analysis between patients treated with or without concurrent chemotherapy resulted no significant differences in treatment outcome. The imbalance in treatment modalities but similar outcome suggested that concurrent chemotherapy in the setting of conformal radiation therapy using IMRT after neoadjuvant chemotherapy might not provide further therapeutic value, even in patients with more advanced disease and poor prognostic factor (i.e., male gender).

Clearly, further investigation, preferably in prospective fashion, on the efficacy of chemotherapy delivered concurrently with IMRT for locoregionally advanced NPC is needed. A prospective randomized study is warranted to confirm the additional therapeutic value of chemotherapy when used with IMRT for patients with locoregionally advanced NPC. Such endeavor is not possible without multi-institutional efforts. Moreover, locoregionally advanced NPC include a heterogeneous group of patients with T2b-T4N0M0 and anyTN1-3M0 diseases. Differences in biological behavior between similarly staged diseases, such as T4N0M0 (stage IVA) versus T1N3M0 (stage IVB) are expected. In an insightful editorial, Cooper generated three hypotheses on chemoradiation therapy for NPC: 1. Concurrent chemoradiation therapy might be an overtreatment for stage II NPC; 2. Fine-tuning of the current treatment strategy is needed for stage III NPC; And 3. More effective systemic therapy is needed for stage IV NPC [21]. The results of our series indicated that these hypotheses needed to be addressed especially in the IMRT era. Our sample size is too small to perform subgroup analyses according to T- and N-classification as well as staging groups of the disease. Therefore, whether concurrent chemotherapy could be substituted with neoadjuvant chemotherapy or omitted in certain subgroups or all subgroups of advanced NPC is subject to further investigation. Our results demonstrated that distant metastasis was the major manifestation of treatment failure, and lymph node status was the only significant prognostic factor for metastatic-free survival and overall survival. In addition, results from randomized trials and meta-analyses showed that no effect of adjuvant or neoadjuvant chemotherapy on the long-term survival could be demonstrated [5,17]. These findings emphasized that more effective chemotherapy agents and regimens are urgently needed for the treatment of subclinical metastatic foci in locoregionally advanced NPC.

## Conclusion

IMRT following neoadjuvant chemotherapy for locoregionally advanced NPC provided favorable outcome in terms of 3-year local/regional control, MFS, DFS, and OS. Our results further suggested that concurrent chemotherapy offered no significant value for further improvement of local and regional control to IMRT following neoadjuvant chemotherapy. In addition, the extent of primary disease measured by T-classification has no significant predictive value for prognoses, whereas N-classification is the only significant prognosticator for both MFS and OS. IMRT following neoadjuvant chemotherapy is a strategy that deserves to be optimized and then tested in a prospective randomized Phase III trial to learn its efficacy in selected subgroups of patients with locoregionally advanced nasopharyngeal carcinoma.

## Competing interests

The authors declare that they have no competing interests.

## Authors' contributions

SL, JL, and HL carried out the review of literature and drafted the manuscript. HL performed the statistical analysis. SL, QC, and JP designed the treatment protocol and the study, and helped to with statistical analysis. SL, JL, and JP participated in the study design, performed the data collection and chart review, and helped with the draft of the manuscript.

## Pre-publication history

The pre-publication history for this paper can be accessed here:

http://www.biomedcentral.com/1471-2407/10/39/prepub
